# Supplementary figures and images for: Development and application of an online tool to quantify nitrogen removal associated with harvest of cultivated eastern oysters
Source: PLoS One. 2024 Sep 6;19(9):e0310062. doi: 10.1371/journal.pone.0310062 (PMC11379275; doi:10.1371/journal.pone.0310062)

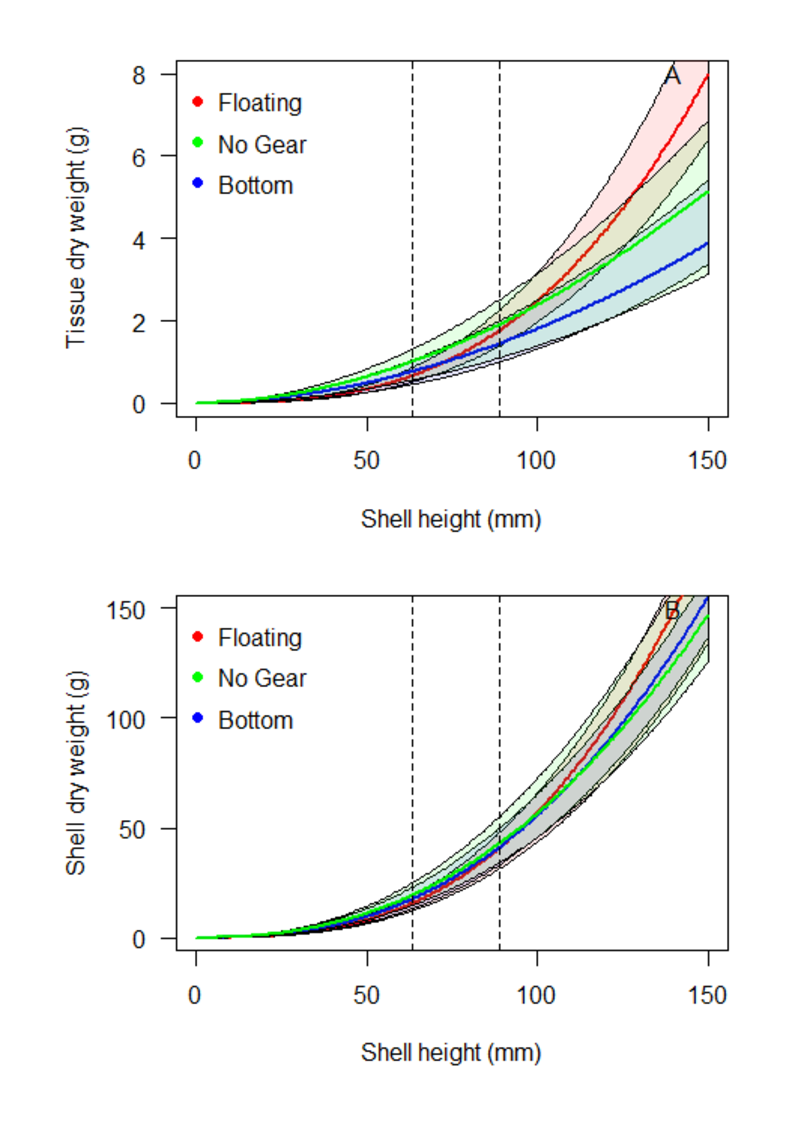

Supplement: S1 Fig — The median values are shown as bold lines. (TIF) [file pone.0310062.s001.tif]
