# Supplementary material for: Development and application of an online tool to quantify nitrogen removal associated with harvest of cultivated eastern oysters
Source: PLoS One. 2024 Sep 6;19(9):e0310062. doi: 10.1371/journal.pone.0310062 (PMC11379275; doi:10.1371/journal.pone.0310062)
Supplement: Attachment — Submitted filename: Rose_ANRC_ response-to-review.docx [file pone.0310062.s002.docx]

Dear Dr. Fernández Robledo,

Thank you very much for the invitation to submit a revised version of our manuscript, “Development and application of an online tool to quantify nitrogen removal associated with harvest of cultivated eastern oysters” as a Tool article type in PLOS One.

We have addressed all journal requirements and responded to each point raised by the reviewers. Our responses are indicated in *blue italicized* text below.

In addition to this rebuttal letter, we include a marked-up copy of the manuscript using the track changes function in Microsoft Word. We also include a clean version of the manuscript in which all changes have been accepted.

We appreciate your continued consideration of our manuscript and new online tool. Please let me know if you have any questions.

Sincerely,

Julie M. Rose, Ph.D.

NOAA Fisheries, Milford Connecticut, USA

Journal Requirements:

1. When submitting your revision, we need you to address these additional requirements.

Please ensure that your manuscript meets PLOS ONE's style requirements, including those for file naming. The PLOS ONE style templates can be found at
<https://journals.plos.org/plosone/s/file?id=wjVg/PLOSOne_formatting_sample_main_body.pdf> and
<https://journals.plos.org/plosone/s/file?id=ba62/PLOSOne_formatting_sample_title_authors_affiliations.pdf>

*We have reviewed the style requirements and file naming conventions and updated our manuscript and figure files to meet all PLOS One guidelines.*

2. Please note that PLOS ONE has specific guidelines on code sharing for submissions in which author-generated code underpins the findings in the manuscript. In these cases, we expect all author-generated code to be made available without restrictions upon publication of the work. Please review our guidelines at <https://journals.plos.org/plosone/s/materials-and-software-sharing#loc-sharing-code> and ensure that your code is shared in a way that follows best practice and facilitates reproducibility and reuse.

*We don’t believe that the findings in our manuscript are dependent on author-generated code, but rather it is a model built on data analysis. We think that we have sufficiently detailed our methods, including R packages used to run the analysis, and we include the regression equation that underpins the model, so that any scientist should be able to take our archived data and easily replicate our results. Please let us know if there are aspects of our code that we should share, and we would be happy to make them publicly available.*

3. Thank you for uploading your study's underlying data set. Unfortunately, the repository you have noted in your Data Availability statement does not qualify as an acceptable data repository according to PLOS's standards.

At this time, please upload the minimal data set necessary to replicate your study's findings to a stable, public repository (such as figshare or Dryad) and provide us with the relevant URLs, DOIs, or accession numbers that may be used to access these data. For a list of recommended repositories and additional information on PLOS standards for data deposition, please see <https://journals.plos.org/plosone/s/recommended-repositories>.

*Our apologies for the confusion! We have used the Zenodo Research Repository, which is on the PLOS One list of approved repositories for data archiving. While the manuscript was in review, we placed our archived data under embargo pending acceptance of this manuscript, which is why the data were not downloadable. We have lifted the embargo and the dataset is freely available at the doi that was originally cited in the text, and our repository should now meet the journal standards for Data Availability. The link to the publicly available data is:* [*https://doi.org/10.5281/zenodo.11966672*](https://doi.org/10.5281/zenodo.11966672)

4. Please review your reference list to ensure that it is complete and correct. If you have cited papers that have been retracted, please include the rationale for doing so in the manuscript text, or remove these references and replace them with relevant current references. Any changes to the reference list should be mentioned in the rebuttal letter that accompanies your revised manuscript. If you need to cite a retracted article, indicate the article’s retracted status in the References list and also include a citation and full reference for the retraction notice.

*We have carefully reviewed the reference list to ensure that it is complete and correct. We are unaware of any retractions of papers cited. There were no changes to the reference list in the revision process.*

**Additional Editor Comments:**

Dear Dr. Rose.

the reviewers have submitted their reviews. Only minor comments. Please pay attention to address Review #2 "I went to the model and found it not so intuitive but I’m an old guy. I got the following error statements on two tries" to make sure the webpage does not give errors.

Regards,

-j

[Note: HTML markup is below. Please do not edit.]

Reviewers' comments:

Reviewer's Responses to Questions

**Comments to the Author**

1. Is the manuscript technically sound, and do the data support the conclusions?

The manuscript must describe a technically sound piece of scientific research with data that supports the conclusions. Experiments must have been conducted rigorously, with appropriate controls, replication, and sample sizes. The conclusions must be drawn appropriately based on the data presented.

Reviewer #1: Yes

Reviewer #2: Yes

2. Has the statistical analysis been performed appropriately and rigorously?

Reviewer #1: Yes

Reviewer #2: Yes

3. Have the authors made all data underlying the findings in their manuscript fully available?

The [PLOS Data policy](http://www.plosone.org/static/policies.action#sharing) requires authors to make all data underlying the findings described in their manuscript fully available without restriction, with rare exception (please refer to the Data Availability Statement in the manuscript PDF file). The data should be provided as part of the manuscript or its supporting information, or deposited to a public repository. For example, in addition to summary statistics, the data points behind means, medians and variance measures should be available. If there are restrictions on publicly sharing data—e.g. participant privacy or use of data from a third party—those must be specified.

Reviewer #1: Yes

Reviewer #2: Yes

4. Is the manuscript presented in an intelligible fashion and written in standard English?

PLOS ONE does not copyedit accepted manuscripts, so the language in submitted articles must be clear, correct, and unambiguous. Any typographical or grammatical errors should be corrected at revision, so please note any specific errors here.

Reviewer #1: Yes

Reviewer #2: Yes

5. Review Comments to the Author

Please use the space provided to explain your answers to the questions above. You may also include additional comments for the author, including concerns about dual publication, research ethics, or publication ethics. (Please upload your review as an attachment if it exceeds 20,000 characters)

**Reviewer #1:** Development and application of an online tool to quantify nitrogen removal associated
with harvest of cultivated eastern oysters
PONE-D-24-24623
As has been discussed in this well written and carefully documented manuscript, the use of shellfish aquaculture provides multiple social, economic and ecological benefits to communities. Among these benefits is the removal of excess nutrient, particularly nitrogen. There are few technologies which address in-water nutrient removal which partially explains the popularity of restorative aquaculture, and shellfish restoration. Coincidentally, commercial aquaculture of oysters has experienced a renaissance in the last decade along the Atlantic seaboard and NGO’s such as The Nature Conservancy are spearheading a global effort for shellfish restoration.
Rose et al. methodically examined the literature and worked with a wide network of shellfish scientists, and federal and state agencies for information regarding biological metrics for oysters grown in aquaculture farms from the states of Maine to Virginia. Using available data on the shell weight, shell size, and % nitrogen in the tissue and shell, the authors have constructed a statistically rigorous application to calculate nitrogen removal from an aquaculture farm which can be used during the aquaculture application process as a part of the list of community benefits. The easy-to-use computer tool will be a boon to those in the process of permitting an aquaculture site.
I believe this manuscript merits acceptance in Plos One. The database underpinning this much needed tool was carefully considered and expertly analyzed by the authors to determine the appropriate relationships between the oyster biological metrics and nitrogen concentrations upon which the equations were developed, and the tool was created. I believe the analyzes were appropriate to address the questions. The tables and figures were very clearly presented.

*Thank you very much for your positive review!*

My few suggestions follow.
With justified text it can be difficult to control spacing, but the authors should check spacing between sentences.

*We have searched the document for instances of double-space and removed two identified.*

L 412 delete ‘a’

*Done*

Table 4 add to caption ‘ploidy, cultivation practices, and regions’

*Done*

L 511 I realize that one of the aims was to create an easy-to-use tool but I am concerned that the difference of nearly 100 lbs of N between triploidy and diploidy is considerable when scaled up. If this tool become widely accepted, is it possible to construct a set of equations for each ploidy?

*One of the advantages of the online tool approach used here is that updates to the calculations are very straightforward when new data becomes available, and research is underway for future expansion of tool functions. Currently, the Chesapeake Bay is crediting triploids at higher nitrogen removal than diploids, based on their literature review from Maryland and Virginia only. This literature did not contain a direct comparison of diploids and triploids grown under identical conditions. A new paper published this year did that comparison, and found only small differences between diploids and triploids on two farms in Virginia and Maryland. These data are included in our nutrient tool and data repository. One additional unpublished dataset from Maine also suggests triploids are not removing nitrogen at higher rates than diploids, and these data are also included here. This is an evolving field in the literature, and until more data across broader geography is available, we feel that it is currently more appropriate to have a single equation for both diploids and triploids. We already have the structure in place in the tool to collect information on ploidy, and the equations can be easily updated.*

L 597 ‘these data’

*Done*

**Reviewer #2:** This ms describes a new synthesis of existing data in the form of development of an online model that predicts nitrogen removal by farmed oysters. If the new synthesis is “original research” then it is appropriate for PLOS ONE. All stats were sensible and interpreted appropriately. Figures 4 – 8 are pretty but most don’t seem necessary for model development? Maybe needed for other reasons? Perhaps move to “Supporting Information”?

*We appreciate the suggestion to streamline the main body of the manuscript. We feel that the figures in question illustrate design choices to simplify the model, and are important visual support for using a single equation across ploidy, cultivation practice, and region. Our preference would be to retain these figures in the main body of the manuscript instead of moving them to Supporting Information.*

It briefly sets the new model in the context of two similar models focused on other geographic areas. I would like to have seen some kind of comparative assessment.

*Thank you very much for this suggestion, we conducted the assessment recommended and have incorporated new text into the Discussion section as follows:*

*“We compared our results to the Florida Shellfish Farm Nitrogen Calculator using inputs of 1,000,000 oysters harvested at a shell height of 3 inches. The Florida Calculator predicted nitrogen removal of 265 pounds, and the ANRC tool predicted 311.6 pounds of nitrogen. We also compared our tissue-only nitrogen removal results to the Chesapeake Bay diploid regression. The Chesapeake Bay diploid tissue regression predicted nitrogen removal of 198 pounds, and the ANRC tool predicted 194.5 pounds.”*

I went to the model and found it not so intuitive but I’m an old guy. I got the following error statements on two tries:

“Warning: Position guide is perpendicular to the intended axis.
i Did you mean to specify a different guide `position`?
Warning: Removed 3 rows containing missing values or values outside the scale range
(`geom_bar()`).”

*We apologize for the error messages! We believe that this error was due to an update to the Posit Connect server, which has since been resolved, and the tool should now be functioning properly. We are unable to replicate any of these errors in the current version of the tool across multiple browsers and on two separate mobile platforms. We plan to roll out the tool to a broad user group of shellfish growers and other industry members in the fall, which should allow us to conduct thorough testing across many platforms, and make updates to user interface based on real-world user feedback.*

Bottom line is: this manuscript describes a valuable contribution (the model) and it should be published, but some revisions are needed.

*Thank you very much for your helpful comments!*

6. PLOS authors have the option to publish the peer review history of their article ([what does this mean?](https://journals.plos.org/plosone/s/editorial-and-peer-review-process#loc-peer-review-history)). If published, this will include your full peer review and any attached files.


If you choose “no”, your identity will remain anonymous but your review may still be made public.


**Do you want your identity to be public for this peer review?** For information about this choice, including consent withdrawal, please see our [Privacy Policy](https://www.plos.org/privacy-policy).

Reviewer #1: No

Reviewer #2: No
